# Alternations of interhemispheric functional connectivity in children with strabismus and amblyopia: a resting-state fMRI study

**DOI:** 10.1038/s41598-021-92281-1

**Published:** 2021-07-23

**Authors:** Jiaxin Peng, Fan Yao, Qiuyu Li, Qianmin Ge, Wenqing Shi, Ting Su, Liying Tang, Yicong Pan, Rongbin Liang, Lijuan Zhang, Yi Shao

**Affiliations:** 1 Department of Ophthalmology, The First Affiliated Hospital of Nanchang University, Jiangxi Center of National Ocular Disease Clinical Research Center, Nanchang, 330006 Jiangxi China; 2 Eye Institute of Xiamen University, Fujian Provincial Key Laboratory of Ophthalmology and Visual Science, Medical College of Xiamen University, Xiamen, 361102 Fujian China

**Keywords:** Neuroscience, Medical research

## Abstract

Previous neuroimaging studies demonstrated that patients with strabismus or amblyopia can show significant functional and anatomical changes in the brain, but alterations of interhemispheric functional connectivity (FC) have not been well studied in this population. The current study analyzed whole-brain changes of interhemispheric FC in children with strabismus and amblyopia (CSA) using voxel-mirrored homotopic connectivity (VMHC).A total of 24 CSA (16 males and 8 females) and 24 normal controls (NCs) consisting of 16 and 8 age-, sex, and education-matched males and females, respectively, underwent functional magnetic resonance imaging (fMRI) scans in the resting state. According to Gaussian random field theory, changes in the resting state FC (rsFC) between hemispheres were evaluated using the VMHC method. The relationships between mean VMHC values in multiple brain regions and behavioral performance were evaluated by Pearson correlation analysis. In contrast to NCs, the CSA group showed significantly decreased VMHC values in the bilateral cerebellum, bilateral frontal superior orbital (frontal sup orb), bilateral temporal inferior(temporal inf),and bilateral frontal superior(frontal sup). CSA have abnormal interhemispheric FC in many brain regions, which may reflect dysfunction of eye movements and visual fusion. These findings might provide insight into the underlying pathogenetic mechanisms of CSA.

## Introduction

Strabismus and amblyopia are common visual developmental disorders characterized by impaired vision and can occur in infancy^1,2^. Strabismus can be divided into comitant and noncomitant forms^3^. It is characterized by abnormalities in eye position and movement, can cause binocular vision impairment, and is often associated with amblyopia and stereoscopic vision loss. Strabismus is an optical manifestation of extraocular muscle discoordination^4^. The prevalence of strabismus in preschool children in eastern China is ~ 5.65%, and ~ 12.8% of the condition is associated with amblyopia^5,6^.

Amblyopia is caused by abnormal visual experiences (e.g., monocular strabismus, anisometropia, and ametropia) during vision development^7^. The prevalence of amblyopia among children in China is 2–3%^8^. It is therefore significant and necessary to explore interhemispheric functional connectivity (FC) in children with strabismus and amblyopia (CSA), which refers to children who have both strabismus and amblyopia.

Magnetic resonance imaging (MRI) techniques have evolved rapidly to provide a non-invasive neuroimaging method that can assess functional and structural changes in the brain^9^. Previous studies demonstrated that interhemispheric synchrony is closely related to visual experience^10,11^. Resting state functional MRI (rsfMRI) is a special technique first proposed by Biswal^12^ that can assess consistent patterns of spontaneous fluctuation of blood oxygen level dependent signals during rest. These signals can be used to measure interhemispheric coordination^13^. rsfMRI and combined studies of functional and anatomic imaging have been applied to various ocular diseases including primary angle-closure glaucoma, congenital comitant strabismus, monocular blindness, and acute eye pain^14–17^.

Quantification of interhemispheric FC between time series at a certain voxel and its mirrored counterpart in the opposite hemisphere can be accomplished with voxel-mirrored homotopic connectivity (VMHC)^18^^.^ This method has been successfully utilized to investigate ophthalmic diseases such as acute open globe injury^19^, retinal detachment^20^,monocular blindness^21^, acute eye pain^22^, corneal ulcer^23^, comitant exotropia^24^, and diabetic nephropathy and retinopathy^25^. However, whether there are interhemispheric FC changes in CSA remains unknown. In the present study, we applied the VMHC method to analyze interhemispheric FC alterations in CSA.

## Materials and methods

### Participants

This study included 24 CSA (16 males and 8 females) who were treated in the Department of Ophthalmology of The First Affiliated Hospital of Nanchang University (Nanchang, China). All of the subjects with CSA (14 with exotropia and 10 with esotropia) met the following criteria: (1) strabismus, (2) greater than one line difference in the best-corrected visual acuity (VA ≥ 0.20 logMAR units) between the amblyopic and fellow eyes, (3) and able to perform center fixation. The exclusion criteria were: (1) children with previous ocular surgery history including intra- and extraocular surgery, (2) other disease (cardiovascular disease, psychiatric disorders, and cerebral infarction), or (3) eye disease (e.g., infection, inflammation, and ischemic disease). In addition, 24 NCs (16 males and 8 females) were matched with the CSA group in accordance with age and sex. The inclusion criteria for NCs were as follows: (1) able to undergo MRI (no pacemaker or implanted metal device), (2) no cardiovascular conditions such as heart disease and high blood pressure, (3) no mental or psychiatric disorders (depression and/or anxiety disorders), and(4) no eye disease history with VA ≤ 0 logMAR units. The study was approved by the ethics committee of the First Affiliated Hospital of Nanchang University, and all methods were applied in accordance with the Helsinki Declaration. The entire study design was provided to the parents of each child involved in the study before they signed informed consent forms.

### MRI parameters

All subjects underwent MRI in a 3-Tesla MR scanner (Trio, Siemens, Munich, Germany). They were required to lie flat on the scanning bed with the head in a neutral position, which was fixed with a foam sponge during the scanning process to prevent head movement. During examination, the subjects remained awake, closed their eyes, and relaxed while avoiding intentional thinking. All scans were performed by the same imaging physician, who observed the subjects until the process was completed successfully. Routine brain localization and T1 and T2 sequences were performed first. The specific parameters were: repeat time = 1900 ms; echo time = 2.26 ms, thickness = 1.0 mm, gap = 0.5 mm, acquisition matrix = 256 × 256, field of view = 250 mm × 250 mm, and reversal angle = 90°. No substantial brain lesions were included in the scanning process. rsfMRI data were acquired using a spoiled gradient-recalled echo sequence. The specific parameters were: repeat time = 2000 ms, echo time = 30 ms, thickness = 4.0 mm, gap = 1.2 mm, acquisition matrix = 64 × 64, flip angle = 90°, field of view = 220 mm × 220 mm. Data were collected continuously at 240 time points, and the scanning range was the whole brain.

### MRI data processing

Data were obtained with functional images through MRIcro software package (www.MRIcro.com) after prefiltering. SPM8 (http://www.fil.ion.ucl.ac.uk/spm) and DPARSFA (http://rfmri.org/DPARSF) software were used to preliminarily analyze data before it was converted to the NIFTI format. Due to possible instability of the initial MRI signal, we removed the first 10 time points of the functional images to ensure the subjects had adapted to the scanning environment. The remaining images were corrected for time differences and small movements, and the signals collected at different times were modified to the same time point. A single T1-weighted magnetization-prepared rapid gradient-recalled echo structure image was transformed into average fMRI data, and then the obtained T1-weighted image was segmented with the DARTEL tool to improve the spatial accuracy of standardized fMRI data. Subjects with head movement > 1.5 mm in the x, y or, z direction or angular rotation > 1.5° were excluded. After correcting for head movement, low-frequency filtering (0.01–0.08 Hz) was modified to eliminate the influence of the physiological high-frequency noises (e.g., breathing and heartbeat). We utilized standard echo planar imaging templates to standardize the fMRI images to the Montreal Neurological Institute space and resampled all voxels to 3 mm × 3 mm × 3 mm resolution.

### Statistical analysis for VMHC

To normalize the data, we transferred the VMHC maps in subjects to z-values with Fisher z-transformations in the REST software (http://resting-fmri.sourceforge.net). The child's brain was used as a mask. Two-sample t-tests were applied to evaluate z-maps in individuals to identify differences in VMHC values between the two groups using global VMHC as a covariate in a voxel-wise manner. Based on Gaussian random field theory (z > 2.3, cluster > 40 voxels, P < 0.01, family wise error [FWE] corrected), we set the voxel level (P < 0.01) as the statistical threshold to make comparisons.

### Brain-behavior correlation analysis

Brain areas with different VMHC values in the CSA group were classified as different regions of interest using REST software. The relationships between mean VMHC values in different brain regions in the CSA group and clinical features were assessed with correlation analysis, with P < 0.01 considered statistically significant.

### Statistical analysis

SPSS version 16.0 (SPSS Inc, Chicago, IL, USA) was used to perform statistical analyses on significant data. With the SPM8 toolkit, differences in VMHC z-maps between the CSA and NC groups were analyzed with two-sample t-tests (z > 2.3, P < 0.01, cluster > 40 voxels, FWE corrected). Pearson correlation analysis was performed to clarify the relationship between mean VMHC values of different brain regions and behavioral performance. P < 0.05 was considered statistically significant.

### Ethics declarations

The study was approved by the ethics committee of the First Affiliated Hospital of Nanchang University and all these methods included have been applied in accordance with the Helsinki Declaration. The entire study design had been provided to the parents of each of the children who involved in the study and parents signed an informed consent form.

## Results

### Demographics and visual measurements

There were no remarkable differences in age (P = 0.732), weight (P = 0.814), or best-corrected VA of another amblyopic eye (P = 0.005) between groups. Details are shown in Table 1.

**Table 1:** The Conditions of participants included in the study. Notes: *P < 0.05 Independent t-tests comparing two groups. *CSA* children with strabismus and amblyopia, *NCs* normal controls, *N/A* not applicable, *PD* prism diopter, *VA* visual acuity, *AE* amblyopic eye, *AAE* another amblyopic eye.

Condition	CSA	NCs	t-value	P-value*
Male/female	16/8	16/8	N/A	> 0.99
Age (years)	9.31 ± 3.16	9.92 ± 2.87	0.273	0.732
Weight (kg)	27.16 ± 7.64	29.53 ± 8.75	0.285	0.814
Handedness	24R	24R	N/A	> 0.99
Best-corrected VA-AE	0.20 ± 0.10	1.05 ± 0.15	− 3.564	0.003
Best-corrected VA-AAE	0.15 ± 0.15	1.05 ± 0.20	− 3.143	0.005
Duration of CSA (years)	9.31 ± 3.16	N/A	N/A	N/A
Esotropia/exotropia Spherical equivalent refractive error (diopters)	14/10 − 1.59 + 0.86 (− 2.25 to 0.50)	N/A − 1.78 + 0.67 (− 2.50 to 1.00)	N/A 0.371	N/A 0.732
Angle of strabismus (PD)	30.15 ± 11.65	N/A	N/A	N/A

### VMHC differences

**Figure 1:**
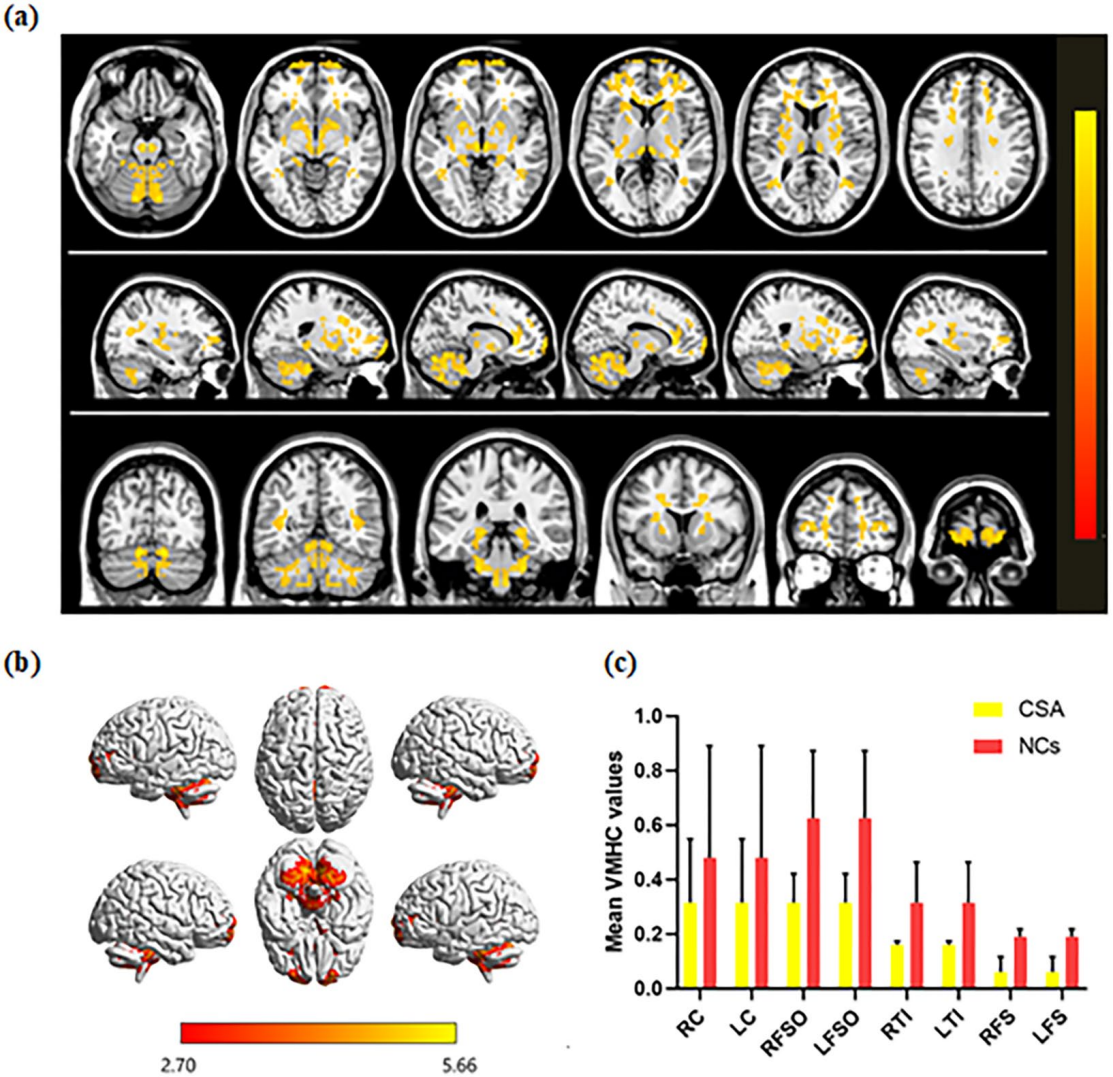
Interhemispheric connectivity in the CSA versus NCs. Significant differences were observed in the RC, LC, RFSO, LFSO, RTI and LTI. (**a**): The lower VMHC values was indicated by yellow areas showed P < 0.01 for multiple comparisons analyze within GRF theory, P < 0.01, z > 2.3, cluster above 40 voxels with corrected FWE; (**b**): The mean VMHC values were altered between the CSA and NCs, P < 0.01 for multiple comparisons using GRF theory (z > 2.3, P < 0.01, cluster > 40 voxels); (**c**): The differences observed in the interhemispheric connectivity were significant in the RC, LC, RFSO, LFSO, RTI and LTI. *RC* right cerebellum, *LC* left cerebellum, *RFSO* right frontal sup orb, *LFSO* left frontal sup orb, *RTI* right temporal inf, *LTI* left temporal inf, *RFS* right frontal sup, *LFS* left frontal sup.

VMHC was significantly decreased in the CSA group compared to NCs in four regions: bilateral cerebellum, bilateral frontal sup orb, bilateral temporal inf, and bilateral frontal sup (Fig. 1, Table 2). The mean VMHC values are represented in (Fig. 1c). In the CSA group, there was no association between clinical features and average VMHC values in different brain regions (all P > 0.05).

**Table 2:** Brain areas demonstrated significantly different VMHC values between CSA and NC group. Notes: The statistical threshold was set at voxel level with P < 0.05 for multiple comparisons using Gaussian random field theory voxels with P < 0.01 and cluster size > 40 voxels, Alphasim corrected. *VMHC* Voxel-mirrored homotopic connectivity, *CSA* children with strabismus and amblyopia, *NCs* normal controls, *BA* Brodmann area, *MNI* Montreal Neurological Institute.

Condition	Left/right	Brain areas	MNI coordinates			Peak voxels	T value
			X	Y	Z		
**NCs > CSA**							
1	Right	Cerebellum	12	− 75	− 21	1751	5.66
2	Left	Cerebellum	− 12	− 75	− 21	1751	5.66
3	Right	Frontal Sup Orb	12	69	− 3	93	4.47
4	Left	Frontal Sup Orb	− 12	69	− 3	93	4.47
5	Right	Temporal Inf	36	− 57	12	108	4.62
6	Left	Temporal Inf	− 36	− 57	12	108	4.62
7	Right	Frontal Sup	21	12	33	58	4.56
8	Left	Frontal Sup	− 21	12	33	58	4.56

### Correlation analysis

**Figure 2:**
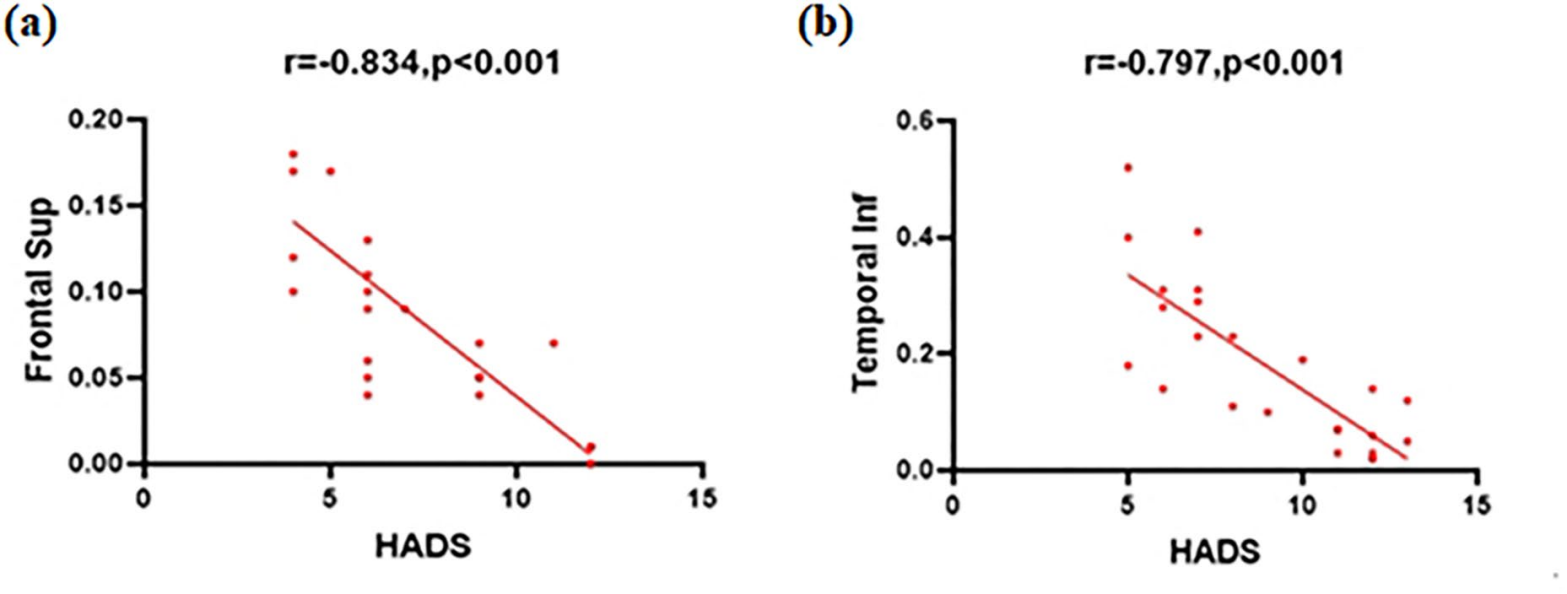
Correlations between the mean VMHC values and the clinical behaviors. (**a**) The HADS scores showed a negative correlation with the VMHC values of the frontal sup (r = − 0.834, p < 0.001), and (**b**) the HADS scores showed a negative correlation with the VMHC values of the temporal inf (r = − 0.797, p < 0.001). *VMHC* voxel-mirrored homotopic connectivity, *HADS* hospital anxiety and depression scale.

In the CSA group, the mean hospital anxiety and depression scale (HADS) scores negatively correlated with VMHC values in the frontal sup (r = − 0.834, P < 0.001), and the HADS scores also negatively correlated with temporal inf VMHC (r = − 0.797, P < 0.001)(Fig. 2).

## Discussion

VMHC is a novel measurement that can reflect changes in interhemispheric FC. The VHMC method has been applied in several ophthalmological diseases (Table 3). To our best knowledge, this is the first time it has been used to study interhemispheric FC in CSA. Compared with NCs, VMHC values were significantly decreased in the bilateral cerebellum, bilateral frontal sup orb, bilateral temporal inf, and bilateral frontal sup in the CSA group.

**Table 3:** Voxel-mirrored homotopic connectivity method applied in ophthalmological diseases.

Author (year)	Disease	(Refs.)
Ye et al. (2018)	Acute open globe injury	^19^
Yuan et al. (2018)	Retinal detachment	^20^
Shao et al. (2018)	Monocular blindness	^21^
Zhang et al. (2018)	Comitant exotropia	^24^
Dong et al. (2019)	Acute eye pain	^22^
Shi et al. (2019)	Corneal ulcer	^23^
Wang et al. (2019)	Diabetic nephropathy and retinopathy	^25^

The cerebellum is located in the lower posterior part of the brain and is involved in maintaining coordinated motor function^26^. Previous studies reported that Purkinje cells, the main output neurons of the cerebellum, can predict eye movements and that the cerebellum helps regulate precise eye movements^27,28^. The cerebellar vermis in particular plays a key role in eye movement^29^. Another group found that the posterior interposed nucleus (PIN) in the cerebellum is critical for coordinated eye movement in strabismic monkeys^30^. Similarly, studies in humans demonstrated that individuals with strabismus exhibit impaired motor behaviors^31,32^. Other groups found that comitant strabismus patients showed low mean diffusivity values in the bilateral cerebellum^33^, and voxel-wise degree centrality (DC) values were decreased in the right cerebellum of comitant exotropia strabismus patients^34^. In this study, the CSA group exhibited reduced VMHC in the cerebellum, suggesting impaired interhemispheric FC function in this brain region. Abnormal interhemispheric FC in the cerebellum might be used as a clinical maker to assess motor control in CSA.

The temporal inf is located in the anterior part of the temporal lobe and manages natural scene coding^35^. This brain region has visual selectivity and responds to three-dimensional structures defined by binocular disparity^36,37^. Temporal inf dysfunction is associated with various diseases including optic neuritis^38^, blindness^39^, and Alzheimer’s disease^40^. Yuan et al. found that patients with mild cognitive impairment had significant regional homogeneity changes in the temporal inf compared to NCs and noted that this brain region plays an important role in multisensory memory and sensory integration^41^. Another group reported altered FC between the visual cortex and right temporal inf in patients with primary open angle glaucoma^42^. Many brain regions are associated with the default model network (DMN), which is activated at rest and disabled during tasks, including the middle frontal gyrus, superior frontal gyrus, inferior parietal cortex, and precuneus^43,44^. It is increasingly evident that the DMN vulnerability plays an important part in depression and anxiety^45^. In line with these findings, the present study showed that CSA had reduced VHMC in the bilateral temporal inf. Moreover, HADS scores were negatively correlated with VMHC values of the temporal inf (Fig. 2b). It may therefore be helpful to assess brain function changes in CSA using the VMHC method. Lower values might reflect the reduction of the role of the temporal inf in the DMN in CSA, who are thought to have dysfunctions in visual selectivity and visual memory.

The frontal orbital cortex is below Brodmann area (BA) 47, which is part of the frontal cortex associated with processing language and grammar^46,47^. In addition, BA 47 is thought to control the perception of musical structure^48^. The patients with the right eye monocular blindness showed lower VMHC values in the superior parietal lobule (BA7)^21^. In the present study, CSA showed remarkably decreased VHMC in the bilateral frontal sup orb, which may be a reflection of impaired language understanding in CSA.

The frontal lobe is located in the anterior central sulcus, and injuries in this region can cause impairment of voluntary movement, language expression, memory. There is a positive correlation between the DMN and cognitive control network in the frontal gyrus^49^. Huang et al. detected alterations in the whole brain microstructure of patients with esotropia using diffusion tensor imaging and found that average diffusion coefficient was markedly reduced in the left middle frontal gyrus^33^. Another group reported decreased voxel-wise DC values in the right middle frontal gyrus were observed in patients with comitant exotropia strabismus^34^. In a former study, patients with corneal ulcer had decreased VMHC values in the medial frontal gyrus^23^. In addition, Yuan et al. found that retinal detachment patients had significantly lower VMHC values in the bilateral occipital lobe^20^. In patients with acute open globe injury, they exhibited reduced VMHC values in the lingual gyrus^19^. Furthermore, the frontal lobe of patients with comitant exotropia showed decreased white matter volumes^50^, and the middle frontal gyrus of amblyopic patients exhibited reduced gray matter density^51^. In the present study, VHMC was decreased in the frontal sup of the CSA group compared to NCs, indicating that this region contributes to SA and is involved in visual processing and associated eye movements (Table 4).

**Table 4:** Brain regions alteration and its potential impact. *CSA* children with strabismus and amblyopia, *NCs* normal controls.

Brain Rigons	Experimental result	Brain function	Anticipated results
Cerebellum	CSA > NCs	Encoding of coordinated motor, the execution of precies eye movements, associated with saccadic eye movements	Impaired motor control function
Frontal Sup Orb	CSA > NCs	Language and grammatical processing	Dysfuction of language understanding
Temporal Inf	CSA > NCs	Part of the default model network, involved in multisensory sensory memory and sensory integration	Depression and anxiety, defects in visual selectivity and visual memory
Frontal Sup	CSA > NCs	Part of the default model network and the cognitive control network	Depression and anxiety, involved in visual processing and associated eye movements

Consequently, we hypothesize that VMHC values in these brain regions might be potential diagnostic markers for CSA. Our study should be interpreted in the context of some limitations. First of all, the sample size was relatively small. In addition, the correlation between clinical characteristics of SA and mean VMHC values require further investigation. Thus, we are looking forward to designing more experiments to further elucidate the underlying molecular mechanisms.

## References

[1] Chen X, Fu Z, Yu J, Ding H, Bai J, Chen J, Gong Y, Zhu H, Yu R, Liu H (2014). Prevalence of amblyopia and strabismus in Eastern China, results from screening of preschool children aged 36–72 months. Br. J. Ophthalmol..

[2] Tarczy-Hornoch K, Cotter A, Borchert M (2013). Prevalence and causes of visual impairment in Asian and non-Hispanic white preschool children, multiethnic pediatric eye disease study. Ophthalmology.

[3] Shi WQ, Zhu PW, Shao Y (2020). The application of functional magnetic resonance imaging in ophthalmology. Am. J. Transl. Med..

[4] Brodsky MC, Fray KJ, Glasier CM (2002). Perinatal cortical and subcortical visual loss, mechanisms of injury and associated ophthalmologic signs. Ophthalmology.

[5] Dickmann A, Petroni S, Alerni A, Parrilla R, Avino G, Battendieri R, Perrotta V, Radini C, Balestrazzi E (2011). Effect of vertical transposition of the medial rectus muscle on primary position alignment in infantile esotropia with A- or V-pattern strabismus. J. AAPOS..

[6] Chan ST, Tang KW, Lam KC, Chan LK, Mendola JD, Kwong KK (2004). Neuroanatomy of adult strabismus, a voxel-based morphometric analysis of magnetic resonance structural scans. Neuroimage.

[7] Farivar R, Zhou J, Huang Y, Feng L, Zhou Y, Hess RF (2019). Two cortical deficits underlie amblyopia, a multifocal fMRI analysis. Neuroimage.

[8] Jin H, Yi JL, Xie H, Xiao F, Wang WJ, Shu XM, Xu Y, Chen S, Ye WX (2011). A study on visual development among preschool children. Chin. J. Ophthalmol..

[9] Brown HDH, Woodall RL, Kitching RE (2016). Using magnetic resonance imaging to assess visual deficits, a review. Ophthalmic Physiol. Opt..

[10] Foubert L, Bennequin D, Thomas MA, Droulez J, Milleret C (2010). Interhemispheric synchrony in visual cortex and abnormal postnatal visual experience. Front. Biosci..

[11] Mima T, Oluwatimilehin T, Hiraoka T, Hallett M (2001). Transient interhemispheric neuronal synchrony correlates with object recognition. J. Neurosci..

[12] Biswal BB (2012). Resting state fMRI, a personal history. Neuroimage.

[13] Fox MD, Raichle ME (2014). Spontaneous fluctuations in brain activity observed with functional magnetic resonance imaging. Nat. Rev. Neurosci..

[14] Huang X, Zhong YL, Zeng XJ, Zhou F, Liu XH, Hu PH, Pei CG, Shao Y, Dai XJ (2015). Disturbed spontaneous brain activity pattern in patients with primary angle-closure glaucoma using amplitude of low frequency fluctuation, a fMRI study. Neuropsychiatr. Dis. Treat..

[15] Gang T, Xin H, Ying Z, Wu AH, Zhong YL, Wu K, Zhou FQ, Shao Y (2016). A functional MRI study of altered spontaneous brain activity pattern in patients with congenital comitant strabismus using amplitude of low-frequency fluctuation. Neuropsychiatr. Dis. Treat..

[16] Li Q, Huang X, Ye L, Wei R, Zhang Y, Zhong YL, Jiang N, Shao Y (2016). Altered spontaneous brain activity pattern in patients with late monocular blindness in middle-age using amplitude of low-frequency fluctuation, a resting-state functional MRI study. Clin. Interv. Aging..

[17] Pan ZM, Li HJ, Bao J (2018). Altered intrinsic brain activeities in patients with acute eye pain using amplitude of low-frequency fluctuation, a resting-state fMRI study. Neuropsychiatr. Dis. Treat..

[18] Zuo XN, Kelly C, Martino AD, Mennes M, Margulies DS, Bangaru S, Grzadzinski R, Evans AC, Zang YF, Castellanos FX (2010). Growing together and growing apart, regional and sex differences in the lifespan developmental trajectories of functional homotopy. J. Neurosci..

[19] Ye L, Wei R, Huang X (2018). Reduction in interhemispheric functional connectivity in the doral visual pathway in unilateral acute open globe injury patients, a resting-state fMRI study. Int. J. Ophthalmol..

[20] Yuan Q, Kang H, Shi W (2018). Disturbed interhemispheric functional connectivity in visual pathway in individuals with unilateral retinal detachment, a resting state fMRI study. Vis. Neurosci..

[21] Shao Y, Bao J, Huang X, Ye L, Min YL, Yang L, Sethi Z, Yuan Q, Zhou Q (2018). Comparative study of interhemispheric functional connectivity in left eye monocular blindness versus right eye monocular blindness, a resting-state functional MRI study. Oncotarget.

[22] Dong ZZ, Zhu FY, Shi WQ, Shu YQ, Chen LL, Yuan Q, Lin Q, Zhu PW, Liu KC, Min YL, Ye L (2019). Abnormalities of interhemispheric functional connectivity in individuals with acute eye pain, a resting-state fMRI study. Int. J. Ophthalmol..

[23] Shi WQ, Liu JX, Yuan Q, Ye L, Su T, Jiang N, Lin Q, Min YL, Li B, Zhu PW, Xu XW, Shao Y (2019). Alternations of interhemispheric functional connectivity in corneal ulcer patients using voxel-mirrored homotopic connectivity, a resting state fMRI study. Acta Radiol..

[24] Zhang Y, Zhu PW, Huang X (2018). Alternations of interhemispheric functional connectivity in patients with comitant exotropia, a resting state fMRI study. Int. J. Clin. Exp. Med..

[25] Wang Y, Wang XY, Chen WZ, Shao Y, Zhou J, Chen Q, Lv J (2020). Brain function alterations in patients with diabetic nephropathy complicated by retinopathy under resting state conditions assessed by voxel-mirrored homotopic connectivity. Endocr. Pract..

[26] Aleh M, Takahashi K, Hatsopoulos NG (2012). Encoding of coordinated reach and grasp trajectories in primary motor cortex. J. Neurosci..

[27] Herzfeld DJ, Kojima Y, Soetedjo R, Shadmehr R (2015). Encoding of action by the Purkinje cells of the cerebellum. Nature.

[28] Nitschke MF, Arp T, Stavrou G (2005). The cerebellum in the cerebro-cerebellar network for the control of eye and hand movements-an fMRI study. Prog. Brain Res..

[29] Hayakawa Y, Nakajima T, Takagi M, Fukuhara N, Abe H (2002). Human cerebellar activation in relation to Accadic eye movements, a functional magnetic resonance imaging study. Ophthalmologica.

[30] Joshi AC, Das VE (2013). Muscimol inactivation of caudal fastigial nucleus and posterior interposed nucleus in monkeys with strabismus. J. Neurophysiol..

[31] Przekoracka-Krawczyk A, Nawrot P, Kopyciuk T, Naskrecki R (1997). Implicit motor learning is impaired in strabismic adults. J. Vis..

[32] Przekoracka-Krawczyk A, Nawrot P, Czaińska M, Michalak KP (2014). Impaired body balance control in adults with strabismus. Vis. Res..

[33] Huang X, Li HJ, Zhang Y, Peng DC, Hu PH, Zhong YL, Zhou FQ, Shao Y (2016). Microstructural changes of the whole brain in patients with comitant strabismus, evidence from a diffusion tensor imaging study. Neuropsychiatr. Dis. Treat..

[34] Tan G, Dan ZR, Zhang Y, Huang X, Zhong YL, Ye LH, Rong R, Ye L, Zhou Q, Shao Y (2018). Altered brain network centrality in patients with adult comitant exotropia strabismus, a resting-state fMRI study. J. Int. Med. Res..

[35] Baddeley R, Abbott LF, Booth MC, Sengpiel F, Freeman T, Wakeman EA, Rolls ET (1997). Responses of neurons in primary and inferior temporal visual cortices to natural scenes. Proc. Biol. Sci..

[36] Verhoef BE, Vogels R, Janssen P (2012). Inferotemporal cortex subserves three-dimensional structure categorization. Neuron.

[37] Janssen P, Vogels R, Orban GA (1999). Macaque inferior temporal neurons are selective for disparity-defined three-dimensional shapes. Proc. Natl. Acad. Sci. U. S. A..

[38] Huang X, Cai FQ, Hu PH, Zhong YL, Zhang Y, Wei R, Pei CG, Zhou FQ, Shao Y (2015). Disturbed spontaneous brain-activity pattern in patients with optic neuritis using amplitude of low-frequency fluctuation, a functional magnetic resonance imaging study. Neuropsychiatr. Dis. Treat..

[39] Yu C, Liu Y, Li J, Zhou Y, Wang K, Tian L, Qin W, Jiang T, Li K (2008). Altered functional connectivity of primary visual cortex in early blindness. Hum. Brain Mapp..

[40] Scheff SW, Price DA, Schmitt FA, Scheff MA, Mufson EJ (2011). Synaptic loss in the inferior temporal gyrus in mild cognitive impairment and Alzheimer’s disease. J. Alzheimers Dis..

[41] Yuan X, Han Y, Wei Y, Xia M, Sheng C, Jia J, He Y (2016). Regional homogeneity changes in amnestic mild cognitive impairment patients. Neurosci. Lett..

[42] Dai H, Morelli JN, Ai F, Yin D, Hu C, Xu D, Li Y (2013). Resting-state functional MRI, functional connectivity analysis of the visual cortex in primary open-angle glaucoma patients. Hum. Brain Mapp..

[43] Garcia A, Luedke A, Dowds E, Tam A, Goel A, Fernandez J (2013). Precuneus volumes and cognitive tests in older adults. Alzheimers Dement..

[44] Liu Y, Li L, Li B, Feng N, Li L, Zhang X, Lu H, Yin H (2017). Decreased triple network connectivity in patients with recent onset post-traumatic stress disorder after a single prolonged trauma exposure. Sci. Rep..

[45] Vicentini JE, Weiler M, Almeida SRM, de Campos BM, Valler L, Li LM (2017). Depression and anxiety symptoms are associated to disruption of default mode network in subacute ischemic stroke. Brain Imaging Behav..

[46] Wright P, Randall B, Marslen-Wilson WD, Tyler LK (2011). Dissociating linguistic and task-related activity in the left inferior frontal gyrus. J. Cogn. Neurosci..

[47] Ahin NT, Pinker S, Halgren E (2006). Abstract grammatical processing of nouns and verbs in Broca's area, evidence from fMRI. Cortex.

[48] Levitin DJ, Menon V (2003). Musical structure is processed in language areas of the brain, a possible role for Brodmann area 47 intemporal coherence. Neuroimage.

[49] Martino J, Gabarrós A, Deus J, Juncadella M, Acebes JJ, Torres A, Pujol J (2011). Intrasurgical mapping of complex motor function in the superior frontal gyrus. Neuroscience.

[50] Yan X, Lin X, Wang Q (2010). DorAl visual pathway changes in patients with comitant extropia. PLoS One.

[51] Xiao JX, Xie S, Ye JT, Liu HH, Gan XL, Gong GL, Jiang XX (2007). Detection of abnormal visual cortex in children with amblyopia by voxel-based morphometry. Am. J. Ophthalmol..

